# Study protocol: Identification and validation of integrative biomarkers of physical activity level and health in children and adolescents (INTEGRActiv)

**DOI:** 10.3389/fped.2023.1250731

**Published:** 2023-09-12

**Authors:** Catalina Picó, Empar Lurbe, Jaap Keijer, Jan Kopecky, Jean-François Landrier, Julio Álvarez-Pitti, Jean Charles Martin, Paula Oliver, Andreu Palou, Mariona Palou, Petr Zouhar, Joan Ribot, Ana M. Rodríguez, Juana Sánchez, Francisca Serra, M. Luisa Bonet

**Affiliations:** ^1^ Laboratory of Molecular Biology, Nutrition and Biotechnology (Group of Nutrigenomics, Biomarkers and Risk Evaluation), University of the Balearic Islands (UIB), Palma, Spain; ^2^ Health Research Institute of the Balearic Islands (IdISBa), Palma, Spain; ^3^ CIBER de Fisiopatología de la Obesidad y Nutrición (CIBEROBN), Madrid, Spain; ^4^ Department of Pediatrics (Innovation in Paediatrics and Technologies-iPEDITEC- research group), Fundación de Investigación, Consorcio Hospital General, University of Valencia, Valencia, Spain; ^5^ Human and Animal Physiology, Wageningen University, Wageningen, Netherlands; ^6^ Laboratory of Adipose Tissue Biology, Institute of Physiology of the Czech Academy of Sciences, Prague, Czech Republic; ^7^ C2VN, INSERM, INRAE, Aix-Marseille University, Marseille, France

**Keywords:** integration analysis, cardiorespiratory fitness, metabolomics, transcriptomics, miRNome, adipokines, cytokines

## Abstract

**Background:**

Physical activity (PA) provides health benefits across the lifespan and improves many established cardiovascular risk factors that have a significant impact on overall mortality. However, discrepancies between self-reported and device-based measures of PA make it difficult to obtain consistent results regarding PA and its health effects. Moreover, PA may produce different health effects depending on the type, intensity, duration, and frequency of activities and individual factors such as age, sex, body weight, early life conditions/exposures, etc. Appropriate biomarkers relating the degree of PA level with its effects on health, especially in children and adolescents, are required and missing. The main objective of the INTEGRActiv study is to identify novel useful integrative biomarkers of PA and its effects on the body health in children and adolescents, who represent an important target population to address personalized interventions to improve future metabolic health.

**Methods/design:**

The study is structured in two phases. First, biomarkers of PA and health will be identified at baseline in a core cohort of 180 volunteers, distributed into two age groups: prepubertal (*n* = 90), and postpubertal adolescents (*n* = 90). Each group will include three subgroups (*n* = 30) with subjects of normal weight, overweight, and obesity, respectively. Identification of new biomarkers will be achieved by combining physical measures (PA and cardiorespiratory and muscular fitness, anthropometry) and molecular measures (cardiovascular risk factors, endocrine markers, cytokines and circulating miRNA in plasma, gene expression profile in blood cells, and metabolomics profiling in plasma). In the second phase, an educational intervention and its follow-up will be carried out in a subgroup of these subjects (60 volunteers), as a first validation step of the identified biomarkers.

**Discussion:**

The INTEGRActiv study is expected to provide the definition of PA and health-related biomarkers (PA-health biomarkers) in childhood and adolescence. It will allow us to relate biomarkers to factors such as age, sex, body weight, sleep behavior, dietary factors, and pubertal status and to identify how these factors quantitatively affect the biomarkers’ responses. Taken together, the INTEGRActiv study approach is expected to help monitor the efficacy of interventions aimed to improve the quality of life of children/adolescents through physical activity.

**Clinical Trial Registration:**

ClinicalTrials.gov, Identifier NCT05907785.

## 1. Introduction

Physical activity (PA) provides significant health benefits across the lifespan and improves many of the established cardiovascular risk factors, such as hypertension, obesity, and diabetes, which have a significant negative impact on overall mortality (1, 2). Although there is strong evidence of the positive relationship between PA and health, it is not very clear what kind and how much PA is necessary, nor can precise individual PA recommendations for the maintenance of health be made. There has been a high heterogeneity both in the definition of PA and its measurement (3). PA level is generally determined from self-report questionnaires, which are subject to potential bias, or through more objective measures, such as pedometers and accelerometers, but there are differences in the way intensity, duration, and frequency of activities are reported that make it difficult to obtain clear, consistent results (2, 3). There is an important need for markers to measure PA more precisely and objectively and, more specifically, markers allowing to relate the degree of PA with effects on the body and health status. PA may produce different health effects depending on type, intensity, duration, and frequency of activities, and also depending on individual factors such as age, sex, body weight, pre-existing health conditions, lifestyle, early life conditions/exposures, etc (4, 5). Not only may such missing biomarkers be a useful tool to measure specific health effects of PA, but they may also be essential for a deeper insight into the complex biological relationships that exist between physical fitness and health determinants.

Most studies dealing with the identification of biomarkers of PA and its relation to health have been performed in adults and athletes [e.g., studies (6, 7)]. Although diseases such as coronary artery disease, hypertension, and type 2 diabetes are generally manifested in adult life, the processes underlying the development of these diseases generally originate in early ages, and childhood and adolescence are critical periods (8). The general WHO recommendation of an average of 60 min/day of moderate-to-vigorous PA in children and adolescents (9) is inversely associated with biomarkers of cardiometabolic risk (10), including lower obesity rates (11). However, globally, it is recognized that children spend an insufficient amount of time in physical activities and too much time in sedentary activities (11). The availability of biomarkers indicative of PA and its relationship with health status (PA-health biomarkers) in these sensitive age groups, will be very useful to make precise, more personalized recommendations to improve health and prevent disease development. The development of PA-health biomarkers will be addressed by the INTEGRActiv study.

Because of the multifaceted nature of homeostasis, omics technologies, such as transcriptomics and metabolomics that broadly analyse functional genomic responses may be particularly valuable for the identification and characterization of biomarkers of PA on health. Peripheral blood cells (PBC) or the subpopulation of mononuclear cells within them (PBMC, including lymphocytes and monocytes) are an attractive source of biomarkers, particularly transcriptomic-based biomarkers because they can be easily obtained by minimally invasive techniques (12, 13). These cells travel through the blood, respond to external and internal factors, and their gene expression profile can partly reflect the expression profile of other tissues and be indicative of metabolic, physiological, and pathological states, as suggested by studies in animal models (14–17) and in humans (18–20). Moreover, PBMC were recently shown to be able to functionally reflect differences in fitness in adults (21). Hence, changes in gene expression in blood cells may inform on physiopathological states and may also have a predictive component (18, 22, 23). The use of PBC as a source of transcriptome markers concerning PA in children/adolescents is almost unexplored so far. In a recent study in children, we have described changes in PBC gene expression associated with low PA, which may be related to the cardiometabolic health effects of PA (24). The INTEGRActiv study will help to validate levels of our previously identified transcripts as indicators of low PA and to identify other potential biomarkers by using targeted and untargeted analyses. Functional links of the encoded transcripts/proteins with cardiovascular and endocrine markers may give information on the effects of PA on body health and will be further explored through literature and data mining/integration.

Among the transcript-based biomarkers, microRNAs (miRNAs) represent specific species of non-coding RNAs that post-transcriptionally regulate gene expression. They are found inside blood cells, but also in the circulation, where they remain stable and can be easily measured (25). PA has been shown to affect levels of specific circulating miRNAs in adult subjects (26), but further research is needed to define them as useful biomarkers. Therefore, circulating miRNAs constitute a new potential regulatory component that may play a role in exercise-induced adaptations and be of interest as PA biomarkers (27, 28). Interestingly, circulating miRNAs also have a great potential as cardiac biomarkers. Therefore, changes in circulating miRNAs could provide further insight into the molecular physiological response to PA, as well as valuable information on the link between PA and cardiovascular health (26, 29, 30) and may thus serve as PA-health biomarkers.

Metabolomics is also a promising approach to exploring the relationship between PA and health, a relationship that can differ between groups and individuals. Extensive plasma metabolite profiling can provide an overview of the metabolism with a level of description that goes beyond genetic information and more closely reflects the phenotype, thus helping to connect genotype to phenotype at the molecular level (31). There is no question that PA affects the metabolome [e.g., (7)], but it is necessary to better understand when these changes occur and for how long they persist. It would also be desirable to identify those biomarkers of PA that indicate and predict metabolic health, including cardiovascular health and propensity to obesity (32). To identify such biomarkers, we will conduct comprehensive untargeted metabolomics and lipidomics analysis on a wide range of low-molecular-weight analytes. In our previous studies in mice within the European project BIOCLAIMS, we learned that plasma acylcarnitines (markers of lipid catabolism) could also serve as a very early and sex-specific biomarker of obesity propensity (33). Here, we will characterize these and other analytes, such as the novel biomarker of PA, N-lactoyl-phenylalanine (34).

Secreted regulatory molecules are also of interest as potential biomarkers and mediators of PA responses. Among them, myokines are signaling molecules released by muscles and involved in the crosstalk of muscle with other organs/tissues (35). Circulating myokines have been associated with muscle contraction and with multiple metabolic effects, such as cardiovascular and anti-inflammatory effects, and the promotion of energy metabolism and “browning” in white adipose tissue, most of them related to the beneficial effects of PA (36). Interestingly, adipose tissue is targeted by myokines and can respond to myokines by changing the production of specific adipose-born cytokines, the so-called adipokines. This myokine/adipokine crosstalk is very relevant in modulating molecular events to ensure whole-body metabolic homeostasis (37). Hence, the study of selected myokines and adipokines is planned as potential PA-health biomarkers.

Thus, the INTREGRActiv study focuses on children and adolescents and will assess PA and relate PA to cardiorespiratory and muscular fitness, measures of body weight and body fat, cardiovascular risk factors and endocrine markers, cytokines, circulating miRNA, gene expression profile in blood cells, and metabolomics profile in plasma to derive new, useful PA-health biomarkers (Integrative biomarkers of PA and health) in children and adolescents. Data regarding perinatal factors, food consumption frequency, sleep behavior, and other lifestyle and social determinants may also have a great impact on health and PA responses and will also be considered.

## 2. Methods and analysis

### 2.1. Aim and setting of the study

The main objective of the INTEGRActiv study is to identify novel useful integrative biomarkers of PA level and its effects on body health (PA-health biomarkers) in children and adolescents and to carry out a first validation stage. The identification of novel candidate biomarkers will be achieved by combining measures of PA and cardiorespiratory and muscular fitness assessment with anthropometric measures, measures of cardiovascular risk factors, endocrine markers, cytokines, and circulating miRNA in plasma, gene expression profile in blood cells, and metabolomics profile in plasma in prepubertal and pubertal children/adolescents in different categories of BMI *z*-score. Besides the influence of BMI, age, pubertal state, and gender, possible confounding factors such as diet and lifestyle-related factors including sleep behaviour will be considered. It is expected that newly identified biomarkers reflecting PA level and its relation with health may guide clinical and nutritional/lifestyle advice and public policies related to endorsing personalized PA, with better adherence and response, to promote health and prevent disease risk factors since early stages of life.

### 2.2. Design of the study

**Figure 1:**
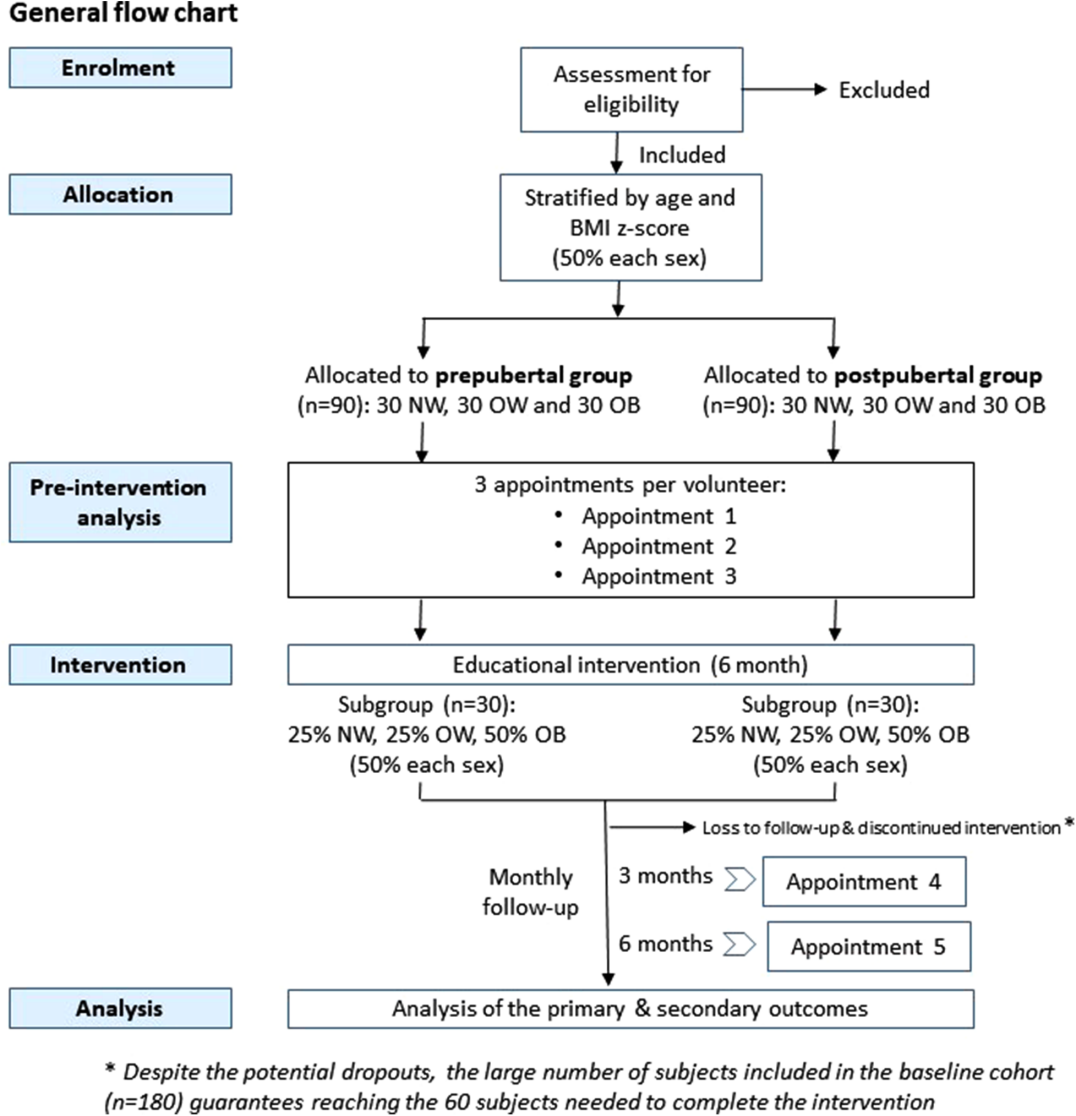
The general flowchart of the INTEGRActiv study.

The INTEGRActiv study is structured in two phases. The first baseline phase (phase 1) is to identify biomarkers of PA and health in a new core cohort of 180 volunteers to be established. In the second phase (phase 2), an educational intervention and its follow-up will be carried out in a subgroup of these subjects (60 volunteers), to allow a first validation step based on the evolution of the identified biomarkers. The general flowchart of the study is illustrated in Figure 1.

### 2.3. Establishment of the core cohort, data collection, and procedures

#### 2.3.1. Participants

A total of 180 volunteers (core cohort) will be recruited and distributed into two age groups: prepubertal individuals (*n* = 90), including children, from 8 to 10 years, and postpubertal adolescents (*n* = 90) from 13 to 15 years. Each group will include three subgroups (*n* = 30) of subjects with normal weight (NW), overweight (OW), and obesity (OB) as defined by WHO criteria (38). Volunteers will be equally distributed between both sexes in each group and subgroup. The sample size of the core cohort is based on our previous expertise in different projects aimed at identifying biomarkers (mainly gene expression biomarkers analysed in blood cells by omics techniques), in which we used even a lower number of volunteers [e.g., studies (24, 39)]. Moreover, the combination/integration of different types of biomarkers that will be performed (based not only on gene expression, but also considering lipidomic, metabolic and miRNA profiles) makes its more likely to be able to detect significant metabolic differences and more robust biomarkers. Additionally, the number of volunteers of the core cohort is also expected to be sufficient to achieve the required sample size for the intervention study, which was calculated as explained in Section 2.4.

Recruitment of children/adolescents will be performed through public advertisement, among the patients of the Cardiovascular Risk Unit of the Pediatric Department of the General University Hospital of Valencia (Spain), among patients of primary care pediatricians, as well as in recruitment campaigns in sports academies and summer camps. Children/adolescents will be informed of the study, and those who express interest and meet the specific requirements of the different groups in the core cohort will be selected for recruitment. Priority will be given to individuals expressing interest in the subsequent intervention to guarantee the minimum number of volunteers required. Pubertal assessment will be performed by experts in pediatric endocrinology. Children/adolescents with regular medication, reported pathologies, and/or the presence of eating disorders will be excluded to avoid potential confounding factors. Individuals with clinical recommendations against the performance of PA will be also excluded. Screening of eating disorders will be done using the SCOFF and DEBQ questionnaires (40, 41). During recruitment, all volunteers will be informed that they will have the possibility to participate in a 6-month duration educational intervention (see below).

#### 2.3.2. Data collection and procedures

Each volunteer will be given three initial appointments to obtain relevant information and blood samples, and to perform different procedures (detailed information of the procedures developed in each appointment is provided in Table 1).

**Table 1:** Information of the procedures developed in each appointment. .

Phase of study	Procedure
Baseline -core group *(App. 1, 2 & 3)*	Weight, height and body composition
	Blood pressure measurement
	Blood sample collection
	Blood sample processing
	Medical history and physical examination
	Cardiorespiratory Fitness assessment
	Online questionnaires: KIDMED, PAQ C/A, Sedentary-screentime Q, PSQ, SCOFF, DEBQ (OLQ)
	Muscular fitness assessment
	Objective assessment of PA and gait
	Data recovery and analysis
	Accelerometer removal
	Dietary intake and PA assessment
	Medical Intervention
3-month follow-up *(App. 4)*	Weight, height and body composition
	Blood pressure measurement
	Medical Follow-up
6-month follow-up *(App. 5)*	Weight, height and body composition
	Blood pressure measurement
	Blood sample collection
	Blood sample processing
	Medical history and physical examination
	Online questionnaires: KIDMED, PAQ C/A, Sedentary-screentime Q, PSQ, SCOFF, DEBQ (OLQ)
	Objective assessment of PA and gait
	Data recovery and analysis
	Accelerometer removal –after 7 days–

Each volunteer will be given three initial appointments to carry out the different proofs/questionnaires and obtain required biological samples. Those volunteers who participate in the intervention will be given two additional appointments. App., appointment.

##### 2.3.2.1. Anthropometric parameters

Weight, height, and waist circumference will be measured by trained nurses to derive BMI, the corresponding standard deviation (SD) and BMI Z score, and waist-to-height values as useful indicators of abdominal obesity and cardiometabolic risk in children. Subjects with a BMI Z score below 1 will be classified as presenting normal-weight; those ranging from 1 to 2 will be defined as subjects with overweight, while obesity is defined by a BMI + 2 SD value (38). Waist circumference will be measured at the midpoint between the iliac crest and the costal margin at the midaxillary line in the standing position at the end of a gentle expiration, and waist percentiles will be calculated according to growth curves (42).

##### 2.3.2.2. Sociodemographic and clinical variables/information

Data regarding perinatal factors (e.g., birth weight and birth complications), breastfeeding duration, weight gain during the first 5 years of life, eating habits during childhood and feeding difficulties, assessment of pubertal stage, and date of menarche and menstrual cycles (in women) will be collected through a clinical interview. Personal and family health history, leisure time activities, family structure and peers, school setting, family affluence, social inequality, and parental weight/height and age will also be recorded.

##### 2.3.2.3. Questionnaires on dietary intake, PA, sedentary and sleep habits

Dietary intake will be assessed by an expert nutritionist following the Guidance on the EU Menu methodology (43); adherence to the Mediterranean diet will be evaluated using the KIDMED questionnaire (44, 45). PA level will be evaluated using the PA Questionnaire for Older Children and Adolescents (PAQ-C, PAQ-A), which has been previously used to classify them into different activity levels (46, 47) and to investigate the PA and health outcomes relationship (46, 48). Sedentary and screen time (49) and sleep habits (50) will be assessed using validated questionnaires. All questionnaires are in Spanish. For each participant, questionnaire responses will be completed online (with the support of parents and investigators) and automatically recorded in the software database (Lyme Survey).

##### 2.3.2.4. Blood sample collection

PBC will be obtained via antecubital fossa venipuncture in the early morning, under fasting conditions. Blood samples will be collected in PAX tubes, to allow optimal conservation and extraction of RNA from PBC for gene expression analyses, and in tubes containing or not anticoagulants (heparin) to obtain plasma and serum, respectively, for analyses of biochemical parameters, miRNA, cytokines, and metabolomics. Circulating biochemical parameters analyzed will include those classically related to metabolic syndrome; abnormal values will be defined from normative data (51).

##### 2.3.2.5. Blood pressure (BP) measurements

BP will be measured three consecutive times by nurses using a validated monitor (Omron-M705) oscillometric recorder, according to the European Society of Hypertension (ESH) Guidelines (52). Volunteers will be classified as normotensive, high-normal, stage 1, or stage 2 hypertensive according to the criteria of the ESH Guidelines, in which the fourth report normative values were used for interpretation in adolescents <16 years (52).

##### 2.3.2.6. Cardiorespiratory fitness (CRF) assessment

CRF refers to the capacity of the circulatory and respiratory systems to supply oxygen to skeletal muscle mitochondria for energy production needed during PA. Low or unhealthy CRF is a strong, independent predictor of cardiovascular disease (CVD) and all-cause mortality in adults (53). In youth, CRF is a predictor of several health indicators, including cardiometabolic health (54, 55), premature CVD (56), academic achievement (57), and mental health (58). CRF will be measured as maximal oxygen consumption (V˙O2max) from cardiopulmonary exercise testing, using a treadmill test and the Balke protocol (59) modified to enhance a secure environment, i.e., avoiding running in steep slopes and taking subjects to exhaustion, especially for the youngest population and those volunteers with moderate or severe obesity (60).

##### 2.3.2.7. Muscular fitness assessment

Muscular fitness will be assessed following the recommendations of the ALPHA Health-Related Fitness Test Battery for Children and Adolescents. The handgrip strength and the standing broad jump tests will be used to assess musculoskeletal fitness (61).

##### 2.3.2.8. Objective assessment of gait and PA

Triaxial accelerometers (Actigraph model wGT3X, Manufacturing Technology Inc, Fort Walton Beach, USA) will be used for objective measurement of PA and to detect gait disturbances. The accelerometer will be fixed at the waist above the right iliac crest. (1) In the laboratory, the subject will be asked to walk on a treadmill for 10 min adjusting the speed to walking pace, without interfering with his/her usual walking pattern. (2) PA will be monitored for 7 consecutive days. The device will only be removed in the case of swimming (this will be recorded through questionnaires). During this period, adolescents who own a mobile phone will be advised to use the Google Fit App to validate their step count accuracy. The software (ActiLife Software) will be used to analyze the signals. Among other variables, the total PA time will be selected, segmenting it into different degrees of intensity (light, moderate and vigorous).

### 2.4. Educational intervention

To validate the biomarkers identified in the first stage of the project, an intervention study will be carried out in a subset of the core cohort. All cohort volunteers and their parents will be invited at enrolment to attend a 1 h educational session conducted by two pediatricians at the hospital. Topics covered will include the importance of weight loss in overweight/obesity and its maintenance, a therapeutic nutritional approach to childhood obesity, and the role of PA in cardiovascular fitness. A subgroup of the core cohort encompassing 25% of the participants with normal weight (NW), 25% with overweight (OW), and 50% with obesity (OB), i.e., 60 volunteers (30 for prepubertal and 30 for postpubertal groups) will be formally included in the intervention. Each volunteer participating in the intervention will be given two additional appointments to perform the different proofs/questionnaires and obtain the required biological samples (Table 1). Each individual will be his/her control, so no additional control group is required, and the results will be analyzed individually by performing a pre-post intervention statistical analysis. The number of participants to be included in the educational intervention study has been calculated to achieve a significant 0.2-point BMI *z*-score reduction between the estimated mean and the sampling mean, with a statistical power of 80% and an alpha risk of 0.05 in a one-sided test. This reduction in BMI *z*-score has been associated with a clinically significant improvement in children and adolescents with overweight (62). Potential abandonments will be compensated by incorporating more subjects from the core cohort, which is feasible since educational intervention is offered to all subjects from the core cohort (*n* = 180). A similar sample size has been used with success in previous intervention studies (63, 64).

The educational intervention proposed is based on the PAIDO Programme (www.programapaido.es), an outpatient family-based multidisciplinary programme that combines PA, education on nutrition, and behavioral therapy (63). It is a 6-month intervention aimed to improve PA and dietary habits and thus an improvement of metabolic health and body weight loss is expected, particularly in the OW and OB groups. This intervention has demonstrated efficacy in improving body composition and cardiovascular fitness (63, 64). The dietary intervention will be focused on the promotion of the Mediterranean diet as a healthy nutritional recommendation, with follow-up and advice from a dietitian-nutritionist. Participants will be encouraged to reduce sedentary behavior (watching television, playing computer games, playing board games). The performance of aerobic and strength physical exercises will also be scheduled, progressively increasing the objectives. During the intervention, children, adolescents, and their parents will participate in 6 (online and on-site) sessions (1 per month). At 3 months after the initial evaluation, volunteers of the intervention study will be re-evaluated (body composition) and at 6 months they will be re-sampled (PBC, plasma/serum) and re-evaluated for general parameters (body composition, PA by accelerometry and questionnaires, and KIDMED questionnaire), as above described.

### 2.5. Laboratory procedures—omic analyses

General metabolic assessment and myokines/adipokines, transcriptome, miRNAs, and metabolome analyses will be performed in plasma and PBC samples from subjects of the core cohort during the biomarker generation phase (phase 1) to provide data for subsequent integrative analysis. In particular:

- Glucose, insulin, lipid profile (total, LDL, and HDL-cholesterol; triglycerides), uric acid and creatinine levels will be evaluated in plasma samples of all subjects of the core cohort using standard automated laboratory enzymatic methods. Insulin resistance will be assessed by the homeostatic model assessment (HOMA) index.
- Plasma levels of circulating myokines and adipokines of special relevance, such as contraction-induced myokines, myokines involved in muscle-adipose tissue crosstalk, and main regulatory adipokines (Irisin, Interleukin-6 and 15, myostatin, FGF21, leptin, adiponectin, etc.) will be measured using commercial assay kits based on the Luminex® xMAP® technology, and ELISA kits in all subjects of the core cohort.
- Whole-genome transcriptomic analysis of PBC samples will be performed by next-generation RNA Sequencing (RNA-Seq) in a representative subset of the core cohort (90 subjects). RT-qPCR analysis of the expression of the most remarkable genes (i.e., those most physiologically plausible and affected) identified in the unbiased transcriptomic study will be used to validate the results in all subjects of the core cohort.
- Combined untargeted and targeted metabolomics and lipidomics analysis of plasma will be performed in all subjects of the core cohort by a LIMeX-4D workflow with 4 different liquid chromatography–mass spectrometry (LC-MS) based methods to annotate a wide range of complex lipids, polar metabolites, and exposome compounds (mainly food components and drugs) (65, 66). Promising biomarkers originating from untargeted metabolomics/lipidomics will be quantified using the reference standardization approach (67).
- For circulating miRNA analysis, RNA will be extracted from plasma samples using the RNAeasy serum/plasma Kit (Qiagen). A miRNome analysis (around 2000 miRNAs) will be assessed in plasma samples of a representative subset of the core cohort (15 of each of the 6 groups), using miRNA sequencing. The most remarkable miRNAs identified (up to 20) will be analysed in the whole cohort by RT-qPCR.

Candidate biomarkers emerging from phase 1 will be measured in plasma and PBC of the subjects in the educational intervention (phase 2) study, at the end of it, using ELISA, RT-qPCR, or biochemical assays, depending on the marker, as above described.

### 2.6. Data analysis and integration

Data will be expressed as mean ± standard deviation (SD). Data will be checked for normality using Shapiro–Wilks normality test and log-transformation will be applied when required. For multi-group comparisons depending on PA levels, body composition, or other health-related parameters available, Levene's test will be performed to assess whether the variance is equal between groups; if the variance is heterogeneous, data will be log-transformed before analysis. Regression models will be applied to analyse the complex data. Comparisons between two groups will be assessed by the appropriate test (*T*-test or the non-parametric Mann—Whitney *U*-test). When appropriate, two-way ANOVA will be used to further determine the effects of different factors, in the case of independent categorical variables. ANCOVA analyses including variables such as BMI, age and gender as covariates will be also used, when appropriate. The threshold of significance will be defined at *P* < 0.05. Correlation analyses are also envisaged to study the possible relationships between the candidate biomarkers and PA levels and other health-related parameters. SPSS and R software will be used for general statistical analyses.

Omics data from transcriptomics and miRNomics (subset of the core cohort) will be subjected to an exploratory analysis of data through principal component analysis (PCA) and partial least squares discriminant analysis (PLS-DA) (when appropriate). Analysis of differential gene expression will be performed through *T*-tests, such as Student's or limma, ANOVA, and false discovery rate (FDR) correction. Gene set enrichment analysis and functional and pathway analyses will be performed. Metabolomics datasets will also be processed using PCA and PLS-DA, and metabolite network mapping using KEGG reactant pairs along with Tanimoto similarity.

#### 2.6.1. Integrated analysis

Data from the PA endpoints (PA, muscular fitness, and CRF describing variables) collected for each individual will be mapped in a principal component analysis plot. Hierarchical cluster analysis will be applied to the PCA scores in order to extract 3 clusters corresponding to low, medium, and high PA scores. The significant differences between the group of individuals will be challenged in a supervised partial-least square discriminant analysis (cross-validation ANOVA and permutation test). All the collected biological data, whether clinical or omics, will be examined in each of the 3 groups of individuals defined therefrom (low, medium, and high PA outcomes). Variables selection to fit the class assignment will be performed independently for each kind of variable with a sight of sparsity. PLS-discriminant analysis (orthogonalized or not), sparse-PLS, and machine-learning methods such as random forest will be applied and resilient variables, selected with all the methods, retained. Statistical models will be built on 4/5 of the population, and their performance assessed by predicting the 1/5 unknowns. This will be iterated 10 times. Complementary AUROC analyses will confirm biomarkers selection robustness. The most resilient variables (expected ∼20 variables from any origin) will be saved, and computed in a final PLS-discriminant analysis. From there, an equation calculated from the beta score of the PLS algorithm and the variable values will be established (using the NIPALS algorithm) to give each individual a score value (with 95% confidence intervals) that fits its PA class (e.g., low, medium, high). This equation will be challenged in the subgroup of the core cohort after the educational intervention. Also, the predictive robustness of the individual score value will be challenged to identified confounding factors (BMI, age, pubertal state, and gender, diet and lifestyle-related factors including sleep behaviour) in a logistic regression model.

After biomarkers discovery, the same methodology as above will be used to identify the biological variables that are related to each PA group, but with much less sparsity. This will allow to examine the molecular regulations that occur at the pathway level. Multi-block PLS method will determine the most influencing kind of variables (miR, transcriptomics, lipidomics, metabolomics, clinics) on group assignment. Pathway enrichment analyses will be also used to provide a biological explanation output. Some focus on specific pathways using in silico reconstituted pathway gathering several omics levels could be also performed (ingenuity pathway analysis, MetExplore, and/or Metscape plugin of cytoscape).

## 3. Discussion

The INTEGRActiv study will address the scientific and societal challenge of identifying integrated markers reflecting both PA level and health (PA-health biomarkers) in children and adolescents, who represent an important target population to address personalized interventions to improve future metabolic health at a societal level. Implementation of the use of the biomarkers relating PA level and health to be identified in the INTEGRActiv study will help the objective assessment of the actual benefits of public health education interventions and PA programs addressed to children and adolescents that are often developed through schools and the primary health care systems. It can also help identify non-responders requiring special attention so that PA programs more adapted to their characteristics can be recommended. Such public programs are urgently required because unhealthy PA habits among children and adolescents are very prevalent in many countries, going hand-in-hand with a high prevalence of overweight and obesity, and there is the awareness that good physical (and nutritional) habits need to be promoted especially in children and youths for their incorporation into their lifestyle, hence ensuring better adherence throughout life. Furthermore, appropriate levels of PA in these critical stages of development might also be required to ensure a proper biological metabolic programming of long-term health.

All in all, the INTEGRActiv study is expected to encompass relevant progress beyond the state-of-the-art for (a) the definition of potential biomarkers for PA level in childhood and adolescence; (b) the provision of relevant mechanistic information for the link between PA and metabolic health in young subjects; (c) the identification of factors such as age, gender, body weight, sleep behaviour and pubertal status that quantitatively affect biomarker responses; (d) the use and development of new tools in biomarker research, including integrative analysis; (e) further assessment and first-step validation of promising candidate biomarkers in an intervention study. It is expected that newly identified robust biomarkers reflecting PA level and its relation with health (PA-health biomarkers) will guide nutritional/lifestyle and clinical advice and public policies related to endorsing biomarker-based personalised PA, with better adherence and response, to promote health and prevent disease risk factors, starting in the early stages of life.

## Ethics statement

The studies involving humans were approved by Ethics Committees of the Consorcio Hospital General Universitario de Valencia (registry number 2/2022). The studies will be conducted in accordance with the local legislation and institutional requirements. Written informed consent for participation in this study will be provided by the participants’ legal guardians/next of kin.
